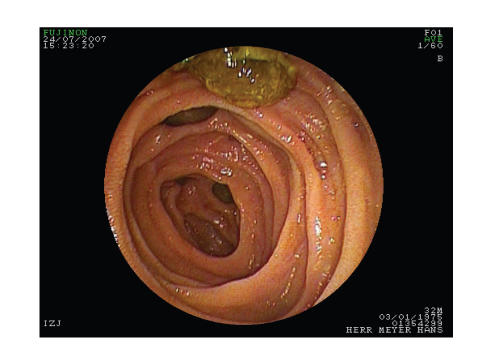# Bleeding Jejunal Diverticulosis in a Patient with Myasthenia Gravis

**DOI:** 10.1155/2008/156496

**Published:** 2008-03-11

**Authors:** I. Zuber-Jerger, E. Endlicher, F. Kullmann

**Affiliations:** Department of Internal Medicine I, School of Medicine, University of Regensburg, 93042 Regensburg, Germany

## Abstract

A seventy-year-old male presented with severe myasthenia gravis and an episode of obscure bleeding. There was a history of gastric ulcer leading to Billroth II surgery twenty-five years ago. Upper endoscopy revealed no pathology. Colonoscopy showed a few solitary diverticula and traces of old blood in the terminal ileum. Capsule endoscopy pictured red smear in the upper jejunum. Diverticula were seen as well. Suspecting bleeding jejunal diverticulosis double balloon enteroscopy was performed. The complete jejunal ascending loop and about 100 cm of the jejunum through the descending jejunal loop could be inspected. Large diverticula with fecoliths were found in both loops. Bleeding had ceased. The patient was discharged to neurology for optimizing therapy for myasthenia gravis.

## 1. INTRODUCTION

Diverticulosis of the big bowel is a common disorder in elderly patients with a high percentage of clinical symptoms and complications. Contrary to this, diverticulosis of the small bowel is considered a rare clinically silent disease.

We present a case with extensive diverticulosis of the small bowel and discuss it in the light of recent literature.

## 2. CASE REPORT

A seventy-year-old male presented with an episode of obscure bleeding. There was a history of gastric ulcer leading to Billroth II surgery twenty-five years ago. No medication affecting blood coagulation was taken. The patient suffered from a severe myasthenia gravis that was treated with pyridostigmine 360 mg. Extensive examination had been done to exclude neoplastic disease. Family history was negative for any neuromuscular or gastrointestinal disorders, for example, inflammatory bowel disease or diverticulosis. No medication affecting blood coagulation was taken. Upper endoscopy revealed no pathology. Colonoscopy showed a few solitary diverticula and traces of old blood in the terminal ileum. Random biopsies did not show any pathology; especially no histological signs of inflammatory bowel disease were present.

**Figure 1:**
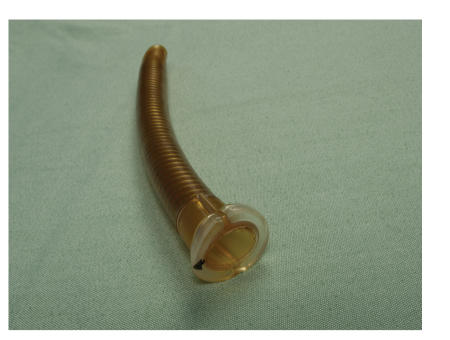


**Figure 2:**
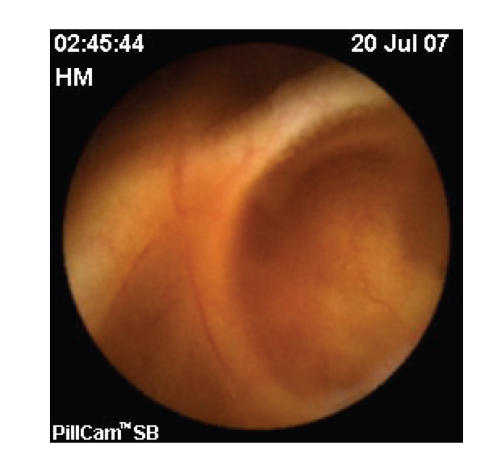


**Figure 3:**
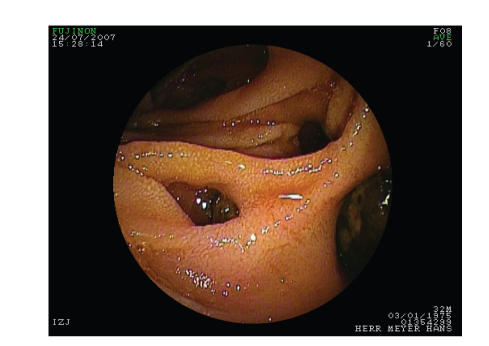


Capsule endoscopy was indicated. The patient required mechanical ventilation and could not swallow the Pillcam capsule. So a flexible tube constructed for the Endocinch system was inserted in the oesophagus (Figure 1). The Pillcam capsule was wrapped in a disposable net and passed endoscopically through the tube into the stomach. Avoiding the ascending jejunal loop the capsule was set free in the descending jejunal loop. The capsule pictured red smear in the upper jejunum. Diverticula were seen as well (Figure 2). Suspecting bleeding jejunal diverticulosis double balloon enteroscopy was performed. The complete ascending jejunal loop and about 100 cm of the jejunum through the descending jejunal loop could be inspected. Large diverticula (Figure 3) with fecoliths (Figure 4) were found in both loops. No biopsy was taken due to risk of perforation. Bleeding had ceased. The patient was discharged to neurology for optimizing therapy for myasthenia gravis. Azathioprin 150 mg was added.

## 3. DISCUSSION

Little is known about diverticulosis of the small bowel. The prevalence of the disorder on autopsy ranges 0.06–1.3%. The disorder seems to be mostly acquired, but two families with extensive jejunal diverticulosis have been published in 1988 and 2007 [1, 2].

Diverticula can occur anywhere in the small intestine, but they are the most common in the jejunum. Jejunal diverticulosis is associated with many diseases, for example, scleroderma, celiac disease, Fabry disease, and Cronkhite-Canada syndrome. An association with myasthenia gravis has not been described so far. Myasthenia gravis has been associated with inflammatory bowel disease [3, 4] and with neoplasia, which were not present in this case. Therapy of myasthenia gravis may have added to the development of jejunal diverticulosis in this patient. Experimental and clinical data have proven that anticholinesterase drugs are responsible for vigorous peristaltic contractions and for an increase of the intraluminal pressure due to muscarinic effects on the smooth muscle of the intestine both in small and large intestine [5, 6].

Usually the disorder is clinically silent. Complications requiring intervention—perforation, bleeding diverticulitis, or intestinal obstruction—occur in 8–30% of patients [7]. Until 1988, 455 cases were published [8]. In 2004, 8 cases were added [9]. Symptoms were noted in 141 cases (29%). Complications requiring surgery were seen in 46 patients (10%). After inflammation (4%) and obstruction (3%) bleeding was the third-most complication (2%). The clinical presentation is usually one of acute massive lower gastrointestinal bleedings comparable to bleeding diverticula of the big bowel. It may be the same pathophysiologic mechanism in most of the cases. In some patients, it may also be due to ectopic gastric or pancreatic mucosa lining a diverticulum [10]. Medication interfering with the blood coagulation system may induce bleeding, too [11].

Bleeding from diverticula of the small bowel may be difficult to localize. The available diagnostic tools are intraoperative enteroscopy, angiography, CT-angiography, multidetector-row CT enteroclysis, wireless capsule enteroscopy, and double balloon enteroscopy.

Visceral angiography is commonly used for identifying the active gastrointestinal bleeding site. A bleeding rate of 0.5 mL/min. is required for diagnosis [12]. Comparison with CT-angiography with the advantage to show not only the vessels but also the surrounding structures demonstrated that only half of the cases seen in angiography is detected in CT-angiography [13].

Wireless capsule enteroscopy is the instrument of choice not only for the overt but also for the obscure gastrointestinal bleeding with a detection rate of the source of bleeding of an average 67% [14, 15]. Comparison with angiography and CT-angiography showed that wireless capsule enteroscopy was superior with a detection rate of 72% versus 56% in angiography and 24% in CT-angiography [13]. When multidetector-row CT enteroclysis with the advantage to show not only the mucosa but also the surrounding structures was compared to wireless capsule enteroscopy it was less sensitive, but was able to detect the source of bleeding in additional 10% cases [12]. In the light of our case, the advantages of multidetector-row CT enteroclysis can be demonstrated. Taking into account the difficulty of placing the capsule and the risk of getting stuck in a diverticulum, we would choose this method retropectively. We would have achieved the same results with multidetector-row CT enteroclysis.

## 4. CONCLUSION

Diverticulosis of the small bowel is a rare disease with a potential for the development of bleeding. Optimal diagnostic tools for this situation should be able to show not only the mucosa but also the vessels and the surrounding structures. CT angiography would fulfil these criteria though it is less sensitive as wireless capsule enteroscopy.

## Figures and Tables

**Figure 4:**